# Supplementary material for: MetaRibo-Seq measures translation in microbiomes
Source: Nat Commun. 2020 Jun 29;11:3268. doi: 10.1038/s41467-020-17081-z (PMC7324362; doi:10.1038/s41467-020-17081-z)
Supplement: Supplementary file 10 — Supplementary Data 7 [file 41467_2020_17081_MOESM10_ESM.zip › File2/Confidence_High_Taxonomy/225972_out.krona.html]

Javascript must be enabled to view this page.

members
magnitude
magnitudeUnassigned
count
unassigned
taxon
rank

225972\_out

5

2
superkingdom
5

976
5
phylum

class
5
200643

5
order
171549

2005525
5
family

genus
5
375288

46503

SRS017521\_contig\_number\_4731SRS020328\_contig\_number\_37924SRS045713\_contig\_number\_21770SRS049402\_contig\_number\_5949SRS140492\_contig\_number\_29233
5
species
